# ‘My Work Matters’: A Qualitative Exploration of Why Staff Love Working in Acute Mental Health

**DOI:** 10.3390/ijerph192013619

**Published:** 2022-10-20

**Authors:** Rachel Mair, Susanna Every-Palmer, Fiona Mathieson, Gabrielle Jenkin

**Affiliations:** Department of Psychological Medicine, University of Otago, Wellington 6242, New Zealand

**Keywords:** mental health, job satisfaction, inpatient care, acute mental health, burnout, psychological

## Abstract

Research findings and media coverage of staff experiences of working in mental health settings tend to focus on the negative aspects of the work such as burnout and stress. These negative aspects affect job satisfaction. Job satisfaction can be understood through the lense of Self-Determination theory, which emphasises the importance of autonomy, competence and relatedness (connection) in job satisfaction. This article reports on staff views on positive aspects of working in acute mental health care, drawing on qualitative interview data collected for a larger study of the social and architectural environment of mental health inpatient facilities in New Zealand. Forty-two inpatient mental health staff participated in semi-structured interviews about their experiences of working in such facilities, sharing the positive aspects of working in this setting, including ‘what they liked most’. Responses were thematically analysed using the Framework Method to identify and organise key themes that were refined iteratively, checking for agreement between researchers. Four key themes were identified: work that matters; the people; the physical and social environment and the extrinsic rewards/personal benefits. The results provide an alternative framing of working in acute mental health settings compared, with commonly reported research findings and media coverage focusing on staff burnout and stress in these settings. Despite the much-documented challenges of working in this often poorly resourced and stigmatized area of health, most participants spoke warmly and enthusiastically about what they did, with frequent use of the word ‘love’ in relation to their work. This was largely because they found the work and social relationships rewarding and they were able to make an important contribution to the wellbeing of mental health service users.

## 1. Introduction

Mental health staff working in adult acute mental health inpatient facilities play a critical role in the recovery of our most vulnerable mental health service users. However, chronic staffing shortages in the mental health workforce add to the already challenging workload and stress of existing staff and affect staff retention [1,2,3]. Understaffing can adversely affect the therapeutic goals of mental health care and contribute to violence in mental health inpatient settings [4]. Staffing shortages and reported negative work experiences are likely to have been compounded by the stressors of the COVID-19 pandemic on the health workforce, particularly for nurses [5,6].

Research and media coverage of the experiences of acute mental health care staff predominantly focuses on the negative aspects of working in acute mental health care, such as burnout, exposure to risk, and stress [7,8,9]. This likely exacerbates the current staffing shortages by adding to the stigma of mental health care work [10]. Research on workplace experiences of mental health staff has been conducted in many countries including Sweden [11]; Finland [12]; Italy [13]; Jordan [14]; Singapore [15]; Australia [16]; the UK [17]; the US [18] and New Zealand [19]. Several of these studies have focused on staff working in acute mental health settings [11,17,20,21].

One theoretical approach to job satisfaction is Self-Determination theory, according to which people need to experience autonomy, competence and relatedness (or connection) to achieve personal growth [22]. Self-determination theory has been well-described in relation to job satisfaction in work settings more broadly [22,23] and has been studied in respect to the work motivation of mental health peer support workers [24]. To the best of our knowledge, Self-determination theory has not previously been examined in terms of the motivations of mental health professionals to choose and continue to work in the acute setting.

Quantitative studies of job satisfaction amongst mental health professionals in a range of settings have reported a range of factors identified as increasing job satisfaction in mental health staff. A Likert scale survey of 118 inpatient nursing staff identified relationships with other staff, recognition and salary as important for job satisfaction [20]. In a study of 121 inpatient and community-based mental health staff, career, working with people, management and money were found to be associated with job satisfaction [17]. A study of 445 community-based mental health staff found job satisfaction to be associated with team role clarity and identification with the team [25]. However, such research is based on quantitative scales that do not allow for in-depth explorations and understanding of why and how these things factor into job satisfaction in the mental health context.

Qualitative research in this area to date focuses on nursing staff and includes semi-structured interviews and focus groups. Based on data from 12 in-depth interviews of mental health nurses in New Zealand, therapeutic relationships, working for the organisation, belonging to a team and maintaining a personal life were found to be key to job satisfaction [26]. An Irish study of mental health nurses across a range of health board settings found that factors influencing levels of job satisfaction predominantly related to the nurses work location, however work routine, off duty/staff allocation arrangements, teamwork and working environment also influenced job satisfaction [27]. However, the findings from these studies may not generalize to the acute inpatient setting.

One Swedish qualitative study was study was specific to job satisfaction in acute mental health inpatient facilities: Semi-structured interviews with 25 nursing staff identified that the meaningful work and good relationships with colleagues, patients and supervisors were important sources of job satisfaction [11]. To our knowledge there are no studies to date on job satisfaction with the full range of clinical staff in acute mental health inpatient facilities.

In the interviews conducted for the current study, our participants described many challenges, including inadequacies in resourcing, poor facilities, short staffing, high demand for services and challenges managing issues such as smoking and aggression. These are reported elsewhere [4,28,29]. The positive side of working in mental health care has received very little attention in the research and literature. Perhaps because it is less newsworthy, it has been overlooked in media reporting of mental health work (i.e., work in acute mental health inpatient settings). We therefore thought it timely to report some of our findings from research based on interviews with staff from four New Zealand acute mental health inpatient facilities. The aim of the analysis was to explore why staff continue to work in such settings, despite the reported negative aspects of working in mental health care.

## 2. Methods

The data for this paper are from a large multidisciplinary study on the social environment of adult acute mental health inpatient facilities in New Zealand that involved multiple data sources and in-depth interviews with staff, service users and family members visiting the facilities [4,28,29]. The original goal of the research was to understand the design and social milieu of the modern acute mental health inpatient facility from the perspectives of people who use them. For this paper, we focus only on the staff interviews as we try to understand why staff like working in these challenging environments.

### 2.1. Data Collection

Data collection for this project required multiple site visits (by author 4), to conduct interviews on inpatient facilities during 2017–2019. Data were collected from a diverse sample of four inpatient units across New Zealand. We maximised case study diversity [30] using building age, condition, and location as criteria. The first four units prioritised for inclusion in our study agreed to participate.

### 2.2. Recruitment

With the help of the management, we invited staff from the full range of occupations on the acute mental health inpatient facilities to participate in our study. Staff were purposively invited to participate to include representation across the following occupations: nurses, nursing care assistants, social workers, occupational therapists, psychiatrists, pharmacists, clinical team leaders, and cultural advisors. The staff participants are described in Table 1.

As shown in Table 1, between 9 and 13 staff interviews were conducted for each facility.

### 2.3. Interview Schedule

The interview schedule asked questions to elicit staff perspectives and experiences across a range of topics chosen to understand the social, therapeutic and architectural milieu of the acute mental health unit. These topics included the Physical Environment (including decoration, furniture, aesthetics and sensory aspects), Therapeutic Environment (available therapies and activities) and Social Organization (unit rules, regimes, social relations and cultural issues). At the conclusion to the questions in the interview schedule, the final question asked was ‘What do you like most about working here?’ This provided most of the data for this analysis. Impromptu data related to positive aspects of working in the acute mental health setting also emerged spontaneously throughout the interview. For this reason, as staff sometimes offered considerable information on positive aspects of working in the mental health setting, it was not always necessary to ask the final question as to what they liked most about ‘working here’. There were slightly fewer impromptu positive comments than comments elicited from the direct question at the end of the interview.

### 2.4. Interviews

All interviews were conducted (by author 4), a social scientist, an experienced qualitative researcher and interviewer. Most took place face to face in the mental health facility, with a few interviews conducted by phone. For practical and budget reasons, the number of interviews conducted on each mental health facility was capped at a maximum of ten each for staff. However, considerable interest in participating resulted in a few additional interviews. Across the four mental health inpatient facilities, 42 interviews were conducted with staff. Interviews lasting around 30–90 min were audio-recorded and transcribed verbatim. Excerpts from the transcripts related to what staff liked about their job were extracted (by author 1).

### 2.5. Analysis

Extracts from the 42 transcripts were analysed thematically aided by the Framework Method to help identify and refine the key themes. The Framework Method is a method of qualitative content analysis. It identifies commonalities and differences in qualitative data, before focusing on relationships between different parts of the data, thereby seeking to draw descriptive and/or explanatory conclusions clustered around themes. While in-depth analyses of key themes can take place across the whole data set, the views of each research participant are not lost [31].

After becoming familiar with the transcripts, the data were coded into themes (by author 1). These codes were then refined through developing a working analytical framework of decided codes by sections with definitions. This analytical framework was then applied to the transcript extracts, with the refined codes being added. This data were then charted into a framework matrix. The data were then interpreted and discussed with the research team. This was an iterative process, with researchers (authors 1, 2 and 4) going back and forth between stages, making suggestions and changes, adding codes and themes, discussing and revising themes, and re-naming themes as agreement was reached as to their key content and meaning.

### 2.6. Reflexivity

This analysis was conducted by an all-female, pākehā (European) team, comprising a social scientist (in her 40’s), two clinicians (a psychiatrist (in her 40’s) and a clinical psychologist (in her 50’s)), and a psychology graduate (in her 20’s). As people who highly value the work done by mental health workforce and who regard mental health as an important public health issue, we wanted to explore why people love working in this area despite the challenges often reported.

## 3. Results

The interviews elicited very rich in-depth data around work and life on acute mental health units in New Zealand. While overall there were many negative comments made about working in these acute mental health settings, as reported previously [4,28,29], despite the many challenges faced by staff working in this setting, there were also many positive comments.

**Figure 1:**
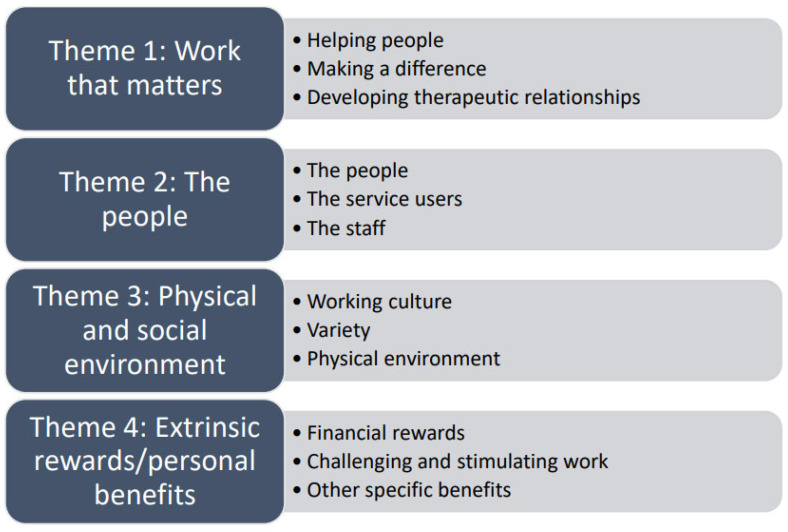
Key Themes.

All staff interviewed could find at least one positive about working in the acute mental health care facility, even if it was just ‘the money’ (as reported by a couple of staff). However, author 4 was struck by how the faces of most staff lit up, with staff becoming animated and enthusiastic when asked what they liked about their work in this setting. Our analyses of their positive comments revealed four key themes that we identified in the data (see Figure 1).

### 3.1. Work That Matters (Theme 1)

Participants frequently discussed elements of their jobs as meaningful and creating positive change. This included **helping people** in multiple ways. This was through improving people’s mental health and offering people support, security, comfort and hope, with results extending to the service user, the service user’s family and the wider community. This included person-centred care, providing pastoral care and being humanitarian in treating people as individuals.

*I like being able to help people, and if it’s just means talking being able to make a small difference or helping them in some small way.*

(C_S_F_NM_003)

*So anything I can do to manaaki [to support, take care of, show generosity, give hospitality to, protect others] those that come into or our unit, I will.*

(D_S_F_M_008)

*I like to offer that message of hope, and I think that first thing I say to people when they come in here is no one lives here forever, so, just to reassure them that no one stays here forever…it’s offering a beacon of hope to people.*

C_S_F_NM_001)

*So that’s one part of my job I quite enjoy, because I am able to give hope to people when they’re in here, because I know how well they can get.*

(B_S_F_NM_008)

*You’re security for them [the patients]...you are a place of safety…Make them [family] know that their family member was in good hands.*

(D_S_F_NM_003)

Participants believed their work **made a difference**. Staff described their work as creating important changes and helping people recover so that they could return to normal function and society.

*I see them [the clients] out in the community from time to time… it’s really cool to see people when they’re feeling well, and things are going well for them.*

(A_S_M_NM_008)

*It’s hard to explain what a difference you can make in just being with somebody when they’re going through what might be one of the worst times of their life.*

(C_S_F_NM_011)

*I wanted to see a difference made for individuals. And I’ve seen that. I’ve seen that with people who have come here, who have been... cycling through. And some of them have actually stopped the admissions, and are moving on a bit more. And I feel that some of the work that I’ve done has actually been helpful to that.*

(A_S_F_NM_004)

As exemplified by the previous quote, staff found it rewarding to be able to support people through a crisis and see their mental health improve such that they could return home. The sense of privilege in being able to do this was a strong and recurrent narrative throughout the transcripts. This brought staff satisfaction and pride, and was a key motivator for remaining in the role.

*Well when there’s success, and when you’ve managed to get a good result for someone.*

(A_S_M_NM_008)

*I love it when people come and who are really unwell...and then, you get to work with them...you know, quite full on until...they can leave...I love that.*

(D_S_F_NM_003)

*I love to see people being brought back down to their baseline, to where they’re able to live a good life again. Yeah, I take pride in my work.*

(B_S_M_NM_006)

Another element of the job that participants enjoyed was **developing therapeutic relationships**. This included talking with the service users, and their families, developing meaningful relationships, building rapport and trust.

*I really like talking with people, and understanding where they’re at.*

(B_S_F_NM_008)

*I’m very much into engaging with people as a fellow—a human being.*

(C_S_M_NM_005)

*You know, it’s like building that that rapport with patients…it takes time, you know… Like they build that trust in it…Even the small conversations like you sitting in there…for a lot of patients that’s like that’s enough.*

(D_S_F_NM_001)

Some participants also mentioned how this staff-service user relationship would grow and become more meaningful when service users would return to the mental health facility. Over time, they came to understand and know these service users better.

*You build up relationships with people that you’ve known for years. People who will always be in the mental health system.*

(B_S_F_NM_009)

*And also like a lot of the tangata whaiora [people who have lived experiences of mental illness and are seeking wellness] I’ve known them, you know, for a long time.*

(D_S_F_NM_001)

*Some patients I get on with really, really well. It’s their nature, really. If they’ve got a good nature, and you can chat away to them…you know when you can talk to someone and when you can’t.*

(A_S_F_M_001)

### 3.2. The Staff, the Service Users and the People (Theme 2)

The second main theme discussed by the participants refers to the people in the mental health inpatient acute facilities, including the staff, the service users and more broadly having a job that involved valued connections with others. There was a strong sense of the inpatient unit as a community, with its own social capital—the networks of relationships among people who cooperate to achieve common goals. When participants were asked what they liked about their work, many responded ‘the staff’, ‘the patients’ or simply ‘the people’.

The first sub-theme is **the people**. Participants often simply talked about ‘the people’ when explaining what they most liked about their job. This could be referring to the staff, the service users, or both. Family and visitors, though also an important part of the mental health facility, were not specifically commented on by the staff in regard to this question.

*I think I’d have to say the staff...and the patients together, really. It’s what makes it.*

(A_S_F_NM_002)

*I get to work with really cool people.*

(C_S_M_NM_005)

The second subtheme is **the service users**. This refers to the service users on the mental health facility being what the staff member liked most about their work.

*Some of the old patients… keep on coming back, you get very fond of them.*

(D_S_F_NM_007)

*My patients. I really like them. Yeah, that’s why I’m still here.*

(C_S_F_NM_010)

Staff elaborated most about their relationships with their colleagues. One common reason of liking **the staff** was the teamwork, referring to the staff working together as a team, rather than individually.

*I think we’ve got a good team…the staff just roll their sleeves up. I mean there’s always people that have issues and stuff,…but at the end of the day, with the limited resources that we’ve got, we still just get on with it.*

(A_S_M_M_007)

*I think all of us as a staff, we all work as a really good team. We work really collectively, and support each other.*

(B_S_F_NM_001)

Another reason referred to was staff relationships. Staff talked about getting on well with colleagues and building up friendships and camaraderie with them.

*I like interacting with some of the staff because I’ve been here such a long time. I’ve grown quite good friendships and we have a lot of fun.*

(D_S_F_NM_007)

*I’ve known quite a few of them for several years, so you build up that friendship and trust.*

(B_S_F_NM_008)

Another reason given for liking the staff was personality attributes of the staff members, with colleagues being described with terms such as ‘nice’, ‘cool’, ‘friendly’ and ‘helpful’.

*I just love everyone here. They’re all so supportive.*

(B_S_F_NM_001)

*The staff…there’s that heart there to help, and to help the people that are in distress.*

(A_S_M_NM_008)

Another reason was that the staff were referred to as committed and hard workers.

*There’s a desire to do the job well.*

(A_S_M_E_005)

Participants also noted that they liked the leadership and management.

*From the management, looking at [manager] and the ACMs, they’re approachable.*

(A_S_M_NM_009)

### 3.3. Physical and Social Environment (Theme 3)

The third theme was the working environment, which included the **working culture, variety** in the work and the people, and the **physical environment** of the mental health facility.

**Working culture** refers to the peer structure and the psychological and social openness of the work environment, such as being a supportive and safety conscious environment, being free to share ideas and ask questions, having a flat hierarchy, and an overall environment of kindness and gratitude. Participants described liking the multidisciplinary collegial environment where people were valued and the working culture was not hierarchical. Each staff member was considered a valuable contributor, irrespective of their position and/or level.

*I like the multidisciplinary part of it…[the] doctors…are no more important than the nurses [who] are no more important than OTs at the end of the day.*

(D_S_F_M_005)

Participants also liked the overall atmosphere or ‘vibe’ around their work.

*I think there’s generally a good vibe within the working experience.*

(A_S_M_E_005)

Participants often loved the **variety** of what they did, whether that be through meeting interesting people, doing different things each day, or variations in the experiences and mental illnesses of the service users. This made their work interesting.

*The diversity of the job. And the people you get to meet…we’ve had lawyers, judges, principals. Mental health will affect everyone and anyone. And that’s what I like. We aren’t just dealing with one type of person.*

(B_S_F_NM_002)

Nought as queer as folk*.

(C_S_M_NM_005)

(* The above quote is a colloquial saying used mostly in the United Kingdom which refers to how people can engage in odd behavior. In this particular interview context, it was used warmly to describe the richness of human diversity and behavior).

Some staff liked elements of the **physical environment** of the mental health inpatient facilities. This theme was specific to staff in the newly built unit, which was seen as a radical improvement on the previous facility, and was not a prominent theme from staff working in the other three units.

*I love the new environment.*

(D_S_F_NM_003)

*Just wow—it’s so beautiful in comparison to the old one, and you just lifted the spirits for whaiora [service users].*

(D_S_F_NM_009)

Only in one other unit was there a positive comment about the physical environment, and this was by a staff member working in the facility located in a park-like setting (the other three were on the hospital grounds).

*I like the light, I like the garden setting between the trees. I’m very lucky. I’ve got this wonderful room…the outlook is great.*

(B_S_F_NM_007)

### 3.4. Extrinsic Rewards/Personal Benefit (Theme 4)

There were also some **personal benefits** that staff often referred to as one of the benefits. This included the **financial rewards.**

*We’re talking about the money…Yeah, it’s very cynical, but no, the money. Time and a half on Saturdays, so it’s worth it.*

(B_S_F_NM_008)

*I’d have to say the money.*

(A_S_F_M_001)

Participants also mentioned they enjoyed the opportunities for mastering new skills, professional development and the **challenge** of the job, as it made each day interesting and motivated them to keep learning.

*And, as well I am constantly learning. I always think, the day I’d say I’ve learned enough, is the day I can go home.*

(C_S_M_NM_005)

*I mean I liked the work, I found that quite challenging. But I also found that quite satisfying.*

(C_S_F_NM_002)

There were **other specific benefits** such as having **flexible work hours**, **gratitude** from service users, getting an ‘ego boost’, the job being fit for their personality and the ‘good days’.

*You know I see patients out in the community and they’re like “oh my God”, you know. They’re like “Remember when you did this for me?” And like because I do it every day. You don’t realise what you do to people but they remember, you know?*

(D_S_F_NM_001)

## 4. Discussion

This study identified and explored the reasons why staff liked working in acute mental health settings, using qualitative in-depth interview data from staff working in a range of occupations in four such New Zealand facilities. We found that, contrary to much of the negative reporting and research evidence on working in this area, many staff spoke warmly and positively about their work in acute mental health services, despite the challenges. In fact, ‘love’ (According to the Collins Co-Build Advanced Dictionary when you love something you like it very much, consider it important and very much want to do it [32]. While participants in this study were not asked to define the term ‘Love’, this may have been what they intended to convey) was a common term used by staff to describe their affection for their work in the mental health care setting. Work that mattered, the people, the working environment and extrinsic rewards/personal benefits were the key themes identified that contributed to job satisfaction.

A number of our results reflect benefits reported in previous quantitative and qualitative studies on job satisfaction for those working in mental health, in particular a study which identified that the important, meaningful work and good relationships with colleagues, patients and supervisors were important sources of job satisfaction in acute mental health staff [11]. The identified importance of financial rewards echoes the results of other studies [17,20].

Our central findings align with Self-determination theory in which people need to experience autonomy, competence and relatedness (or connection) [22] and in particular with a study of job satisfaction from a self-determination theory perspective that found that holding an extrinsic, relative to instrinsic work value orientation was associated with less job satisfaction [33]. While our participants identified extrinsic benefits of their work (such as salary and overtime bonuses), it was the intrinsic rewards that were most emphasised as reasons they loved their job. This is consistent with participants valued being members of a ‘flat hierarchy’ multidisciplinary team which valued independent contributions (aligning with autonomy). They liked mastering skills, the opportunities for professional development and the feeling that they were contributing usefully to an important endeavour (aligning with competence). ‘The people’—the positive connections with other staff and service users, and the ability to connect with experiencing difficult times (aligning with connection or relatedness) was a particularly strong theme.

The qualitative recounting of these motivations may help people considering working in this field to know whether their personal motivations align, and as such, whether they would be able to sustain their enthusiasm for this type of work. Novel findings from our study also include staff reporting the lack of occupational hierarchy as a positive factor in the multi-disciplinary team environment of acute mental health case, liking the management, making a difference, financial rewards, variety, and challenging, stimulating work. In line with our results and self-determination theory, managers and team leaders in acute mental health facilities can likely boost job satisfaction by fostering collaborative working relationships with staff, valuing independent contributions (such as initiatives to improve the ward environment or service user experience), and supporting professional development training that develops interpersonal connection skills.

### Strengths and Limitations

As well as the large size of this study with more than 40 mental health professionals interviewed in-depth, the novelty and multi-disciplinary nature of this study is its key strength. It is rare to find qualitative and rich research on the positives of working in the acute mental health setting. A further strength is the inductive approach taken in this analysis in that it was not theoretically driven, rather we let the data tell the story and direct the theme finding, rather than being constrained by existing theory. We did this by diving into the data before reading and examining the relevant literature and theory. This allowed us to pull out the unexpected findings and key commonalities in the narrative, rather than focusing on extracting evidence to support a particular stance. Another strength of this analysis was the use of investigator triangulation, which allowed for robust discussion on the key themes between researchers of diverse backgrounds (social scientist, psychiatrist, psychologist and psychology graduate). This allowed for attendance to our potential biases in interpreting the findings. Having two researchers independently code the data also added further rigor to the findings. A limitation of this study is the focus on the acute mental health setting, which means the findings are not necessarily generalizable to other mental health settings such as community mental health settings. Additionally, as with all qualitative research, the findings share the limitations of the discipline, including potential selection bias in that the views of those who consented to participate may differ from those who declined.

As well as potentially aiding staff recruitment into acute mental health settings, the results of this study may prove reassuring to service users in that they are consistent with ‘genuine care’ from staff in mental health settings which has been identified as important to service users [34].

## 5. Conclusions

Despite all the negatives reported in the literature and the media around working in mental health, we found more than ‘silver linings’ in the staff narratives. We found genuinely caring and dedicated staff, who worked tirelessly in challenging resource- deprived, stigmatizing settings. Many staff we interviewed revealed many positive aspects of their work and work environment in the acute mental health setting that provide hope for improving recruitment into mental health roles in the future.

This study provides a different angle to the commonly reported research findings and media coverage of burnout and stress for staff working in acute mental health inpatient facilities. Most staff spoke warmly and enthusiastically about what they did, with frequent use of the word ‘love’ in relation to their work. In particular, they identified work that matters, the people, the physical and social environment and extrinsic rewards/personal benefits as important reasons for doing this work. We hope this will be of interest for people considering joining the mental health workforce who might feel apprehensive. Service users should also feel assured of the genuine caring and compassion of many of the staff in acute mental health settings who report loving their work and the people they care for.

These positive findings may also go a small way towards reducing stigma about working in mental health.

## Figures and Tables

**Table 1:** Participant characteristics.

	Unit A	Unit B	Unit C	Unit D	Total	N = Female	N = Māori
*Participants*	9	13	11	9	42	27	7

## Data Availability

The data is not available due to the nature of the qualitative data and the potential for identification of participants.
